# Disability-Free Life Expectancy among People Over 60 Years Old by Sex, Urban and Rural Areas in Jiangxi Province, China

**DOI:** 10.3390/ijerph18094636

**Published:** 2021-04-27

**Authors:** Shengwei Wang, Songbo Hu, Pei Wang, Yuhang Wu, Zhitao Liu, Huilie Zheng

**Affiliations:** 1 Jiangxi Province Key Laboratory of Preventive Medicine, School of Public Health, Nanchang University, Nanchang 330006, China; 411437818006@email.ncu.edu.cn (S.W.); husbo0910@ncu.edu.cn (S.H.); 411437819016@email.ncu.edu.cn (Y.W.); 401440319007@email.ncu.edu.cn (Z.L.); 2 Department of Statistics, University of Kentucky, Lexington, KY 40536, USA; pwa242@uky.edu; 3 Department of Statistics, Miami University, Oxford, OH 45056, USA

**Keywords:** disability, disability-free life expectancy, urban–rural areas

## Abstract

Objective: To estimate and compare age trends and the disability-free life expectancy (DFLE) of the population over 60 years old in 2018 in Jiangxi Province, China, by sex and urban–rural areas. Methods: The model life table was employed to estimate the age-specific mortality rate by sex and urban–rural areas, based on the Summary of Health Statistics of Jiangxi Province in 2018 and the Sixth National Health Service survey of Jiangxi Province. DFLE and its ratio to life expectancy (LE) were obtained by the Sullivan method. Results: In 2018, the DFLE among people over 60 is 17.157 years for men and is 19.055 years for women, accounting for 89.7% and 86.5% of their LE respectively. The DFLE/LE of men is higher than that of women at all ages. LE and DFLE are higher for the population in urban areas than in rural areas. For women, DFLE/LE is higher in urban areas than in rural areas (except at ages 75 and 80). Urban men have a higher DFLE/LE than rural men (except at age 85). The difference in DFLE between men and women over 60 years is 1.898 years, of which 2.260 years are attributable to the mortality rate, and 0.362 years are due to the disability-free prevalence. In addition, the difference in DFLE between urban–rural elderly over 60 years old is mostly attributed to the mortality rate by gender (male: 0.902/1.637; female: 0.893/1.454), but the impact of the disability-free rate cannot be ignored either (male: 0.735/1.637; female: 0.561/1.454). Conclusions: The increase in DFLE is accompanied by the increase in LE, but with increased age, DFLE/LE gradually decreases. With advancing age, the effect of disability on elderly people becomes more severe. The government administration must implement some preventive actions to improve health awareness and the life quality of the elderly. Rural elderly; rural women in particular, need to be paid more attention and acquire more health care.

## 1. Introduction

Continuing evidence suggests that life expectancy is increasing, with a steady declining trend in mortality in both developed and developing countries [1,2,3,4], and the quality of life is becoming a central issue. Do people live more healthily during their long lives, or do they increasingly have sensory, physical, and mental disabilities in their later years [5]? Disability-free life expectancy (DFLE) is the average number of years lived by a person of a particular age without disability [6], which is a combined measure of mortality and disability. The focus of DFLE is on the quality of life, while the life expectancy (LE) is used to measure the quantity of life a person expects to have. LE is also a significant indicator that can be used to allocate resources, measure the success of political programs and understand the changes in the mental, and physical health of the general population [7,8].

Per the Global Burden of Disease (GBD) study [9], the life expectancy of people at birth worldwide was 65.6 years in 1990, and it was 73 years in 2017. In the past 30 years, the life expectancy of the population has increased, which leads to an increase in the elderly population. A greater number of older persons mean more are disabled [5]. Disability brings a great burden of disease and consume a lot of medical and nursing resources. Therefore, it is necessary to calculate DFLE to evaluate the health of the population and provide a basis for policy formulation.

A large number of research studies on DFLE have been carried out in developed countries. Early research into the healthy life expectancy (HLE) focused on disability-free life expectancy for the prevalence of disability, and being a health statistic, it is easy to obtain in most developed countries [10]. At present, relevant research is mainly divided into two categories, one being cross-sectional studies [11,12,13] by using Sullivan’s method and the other one involving longitudinal studies [14,15,16,17,18,19,20] by using the multi-state life table method. To discuss the difference, the population is generally divided into gender or some demographic characteristics such as race, education, or living arrangements. In one of the cross-sectional studies using Sullivan’s method, Gutierrez-Fisac [12] undertook an ecological research study, with the 50 provinces of Spain as the units of analysis. By calculating DFLE for each province based on information from the death registry and the survey on disabilities, impairments, and handicaps, he concluded that both DFLE0 and DFLE65 varied greatly among provinces. Some researchers [12,14,21,22,23] argued that socioeconomic status and education would affect DFLE as well.

In many developing countries, there is a speedy transition from a young age structure to an old age structure. It results in less time for economic development to meet the needs of the elderly in environmental support, social protection, financial security, and health care [24]. In the comparison of China, Ghana, India, Mexico, Russia, and South Africa, India had the lowest DFLE while China had the highest DFLE [25]. The Chinese population is one of the fastest aging populations in the world [5]. In 2018, 17.9 percent of the population were over 60 years old, and the average life expectancy at birth had reached 76.7 years. As the world’s most populous and dynamic society, it is necessary to be concerned about the health quality of the Chinese elderly.

There are three studies of DFLE in China on PubMed. Liu et al. [6] calculated DFLE by region using the Sullivan method; their results showed that the pattern of differences in DFLE by region mirrored the pattern of regional economic development, and that socioeconomic and health care factors partially accounted for these variations. Cheung et al. [26] found that Hong Kong women with more disabilities, tended to live longer than Hong Kong men; it means they had more years of life with disability in their remaining life. Zimmer et al. [27] used eight years of panel data to investigate the association between religiosity (public, private, coping) and DFLE in Taiwan, and found that those who engaged in public and private religiosity lived longer and more disability-free years than others, while DFLE/LE was not different across levels of religiosity.

Currently, there are some arguments that people living in urban areas are wealthier than those living in rural areas, in terms of housing, education, income, health facilities, sanitation, and a lower rate of disability [28]. Islam et al. [7] conducted research on the disability-free life expectancy of the urban–rural elderly in Bangladesh, and they confirmed the above argument and found that there were distinct inequalities in LE, DFLE, and LE with disability (DLE) between rural and urban areas.

Unfortunately, few studies have been conducted in urban–rural dimensions to explore the differences of DFLE in China. China has large health inequalities and regional economic differences, and in a single report of a country’s healthy life expectancy, it is easy to cover up its large regional differences in healthy life expectancy. Besides, there are considerable inequalities in social status, health, and accessibility to health care in China [6]. As the Chinese population ages at an overwhelming speed, it is necessary to study the healthy life expectancy of the elderly in different regions. Jiangxi Province is located in Southeast China and is part of East China, covering an area of 166,900 square kilometers [29]. By the end of 2018, the permanent resident population of Jiangxi reached 46.476 million; the urbanization rate of the permanent resident population was 56% [30], and the GDP was 2198.48 billion yuan; ranking it 16 out of 34 of the provincial administrative regions in China [31]. Based on the above research, we considered selecting the urban–rural elderly over 60 years old in Jiangxi Province, China as the research object. In addition, since most current domestic studies focus on the gender dimension, and no DFLE study had considered both region and gender at the same time, this study aims to investigate the DFLE of the elderly over 60 years in Jiangxi province, which is the least economically developed area in China. The differences between genders (male and female), regions (urban and rural) are discussed. The latest 2018 sixth health services survey data are adopted in this study. These data contain a certain timeline and it is easy to compare with other provinces in China within the same period.

## 2. Materials and Methods

The study proposed in this paper mainly requires the following two kinds of datasets:

The infant mortality rate (IMR) and under 5, mortality rate (U5MR) for each age, sex and administrative region, were selected from the Summary of Health Statistics in Jiangxi (2018); recorded annually by the Health Commission of Jiangxi and used internally. These data came from the maternal and child monitoring system. Based on these data, and after revising the age-specific mortality rate, we used the China model life table [32] to obtain a more accurate life expectancy. This method was constructed using the Murray model life table method [33] based on the census and population sampling data of China.

The disability-free prevalence data were obtained from the Sixth Survey of Health Services in Jiangxi Province in 2018. This survey was a part of the Sixth Health Services survey in September 2018, and was a comprehensive survey to understand the health and health service demand and utilization situation. It has been conducted once every five years. In the Sixth Survey of Health Services in Jiangxi Province, the multi-stage stratified cluster sampling method was adopted to select 3 cities and 3 villages as samples. The sampling unit of the family health survey was households, and 60 households were randomly selected from each sample village (neighborhood committee). The respondents were actual members of the selected households. A total of 2731 elderly people over 60 years old participated in filling in the questionnaire, and 2713 questionnaires were recovered, with an effective rate of 99.3%.

Disability-free life expectancy was calculated by the Sullivan method and the index of health evaluation was the ADL dependence rate. The ADL scale adopted in the sixth health service survey includes 8 items: getting dressed, eating, bathing, getting in and out of bed, going to the toilet, controlling defecation and urination, doing housework, and managing money and property. Each item includes four options: 1—no difficulty; 2—difficulty, but still able to complete independently; 3—difficulty, need help; 4—inability to complete. Subjects who answered 3 or 4 are considered as ADL dependent. The calculation results include LE, DFLE, and DFLE/LE. Z-test was used to compare the differences of DFLE in pairs, and 95% CIs were given. The decomposition method [34] was employed to calculate the DFLE differential contribution years caused by mortality and disability-free prevalence.

## 3. Results

Table 1 shows the results of LE, DFLE, DLE, and DFLE/LE of elderly people over 60 years old, with every 5 years shown as an age group. The life expectancy of men aged 60 in Jiangxi Province, China, is 19.127 years old and that of women is 22.027 years old. Men aged 60 can expect to live 17.157 years without disability, accounting for 89.7% of life expectancy. For women, these figures are 19.055 years and 86.5%, respectively. In this age group, women’s disability-free life expectancy is higher than that of men, but their proportion of life expectancy is lower than that of men. At the age of 85, men have a LE of 5.134 years and a DFLE of 3.496 years. The LE for women is 5.851 years, and they can expect to live 3.060 years without disability. In terms of the DFLE/LE, it drops to 68.1% for men and 52.3% for women in the age 85 group.

**Figure 1:**
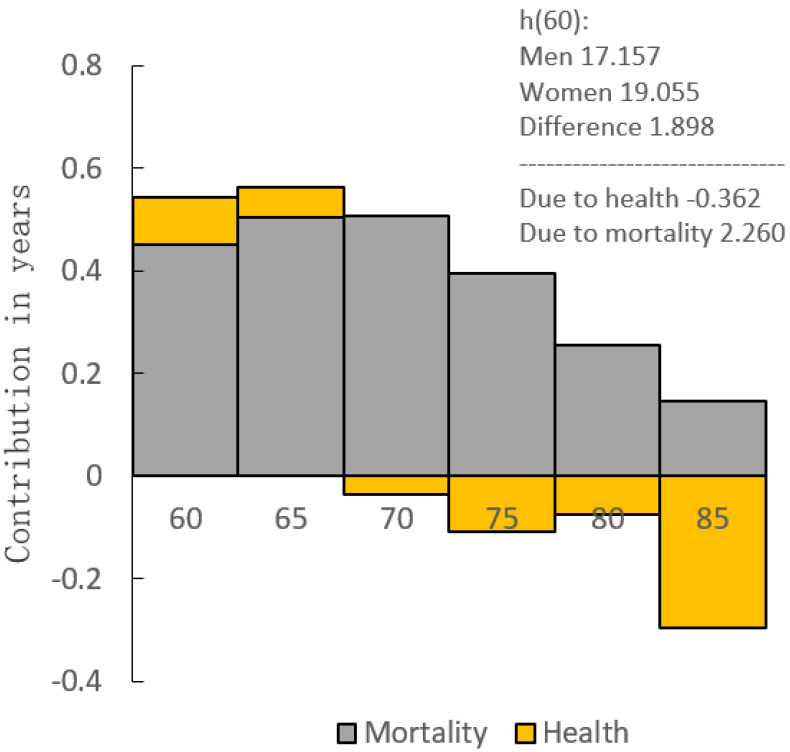
Decomposition of the difference between the DFLE of elderly in Jiangxi Province at age 60 between men and women.

Figure 1 shows that the difference in DFLE between men and women over 60 years is 1.898 years, of which 2.260 years are attributable to the mortality rate, and 0.362 years are due to the disability-free prevalence.

Table 2 divides the total sample into urban and rural dimensions to describe the DFLE of the elderly over 60 in Jiangxi Province. At the age of 60, the LE of urban men is 20.068 years, and the DFLE is 18.258 years while they are 18.957 years and 16.621 years for rural men, respectively. LE and DFLE are 23.102 years and 20.091 years for urban women, and 21.837 years and 18.637 years for rural women. It can be seen from the table that both men and women have higher LE and DFLE in urban areas than in rural areas. Regardless of the region, the LE and DFLE of women is higher than that of men (except in age groups 80, 85). Regardless of sex, the LE and DFLE of urban areas are higher than in rural areas.

**Figure 2:**
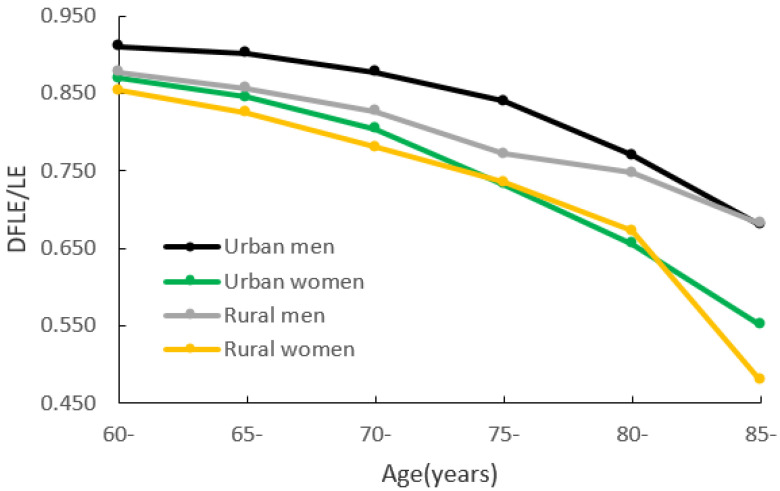
Trends of disability-free life expectancy as a proportion of life expectancy (DFLE/LE) for men and women in urban and rural areas.

Figure 2 shows the trend of DFLE/LE for men and women in urban and rural areas. The DFLE/LE of 60-year-old urban men is 91.0%, and that of urban women is 87.0%, while the DFLE/LE of 60-year-old rural men is 87.7% and that of rural women of the same age is 85.3%. The DFLE/LE of 85-year-old urban men is 68.0%, and that of urban women is 55.0%, while the DFLE/LE of 85-year-old rural men is 68.2% and that of rural women is 48.0%. Both urban and rural men’s DFLE/LE are higher than the women’s at all ages. The ratio of urban men is always higher than that of rural men (except at age 85). For women, the ratio of urban areas is higher than that of rural areas (except at ages 75 and 80).

**Figure 3:**
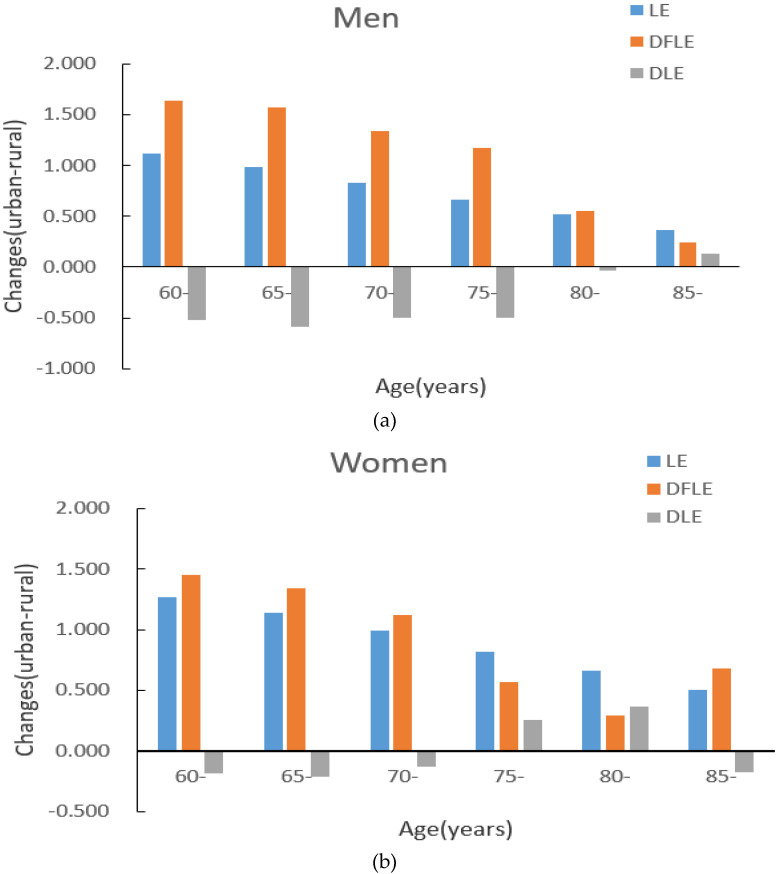
Absolute changes in life expectancy (LE) and disability-free life expectancy (DFLE) between urban and rural areas for men and women. (**a**) men (**b**) women.

Figure 3 shows the absolute change (urban–rural) in LE and DFLE between urban and rural residents. Compared with the LE of urban and rural residents, at the age of 60, the urban men’s is 1.111 years higher than that of rural men, while the urban women’s is 1.265 years higher than that of rural women. At the age of 85, the life expectancy of urban men is 0.367 years higher than that of rural men, and the life expectancy of urban women is 0.504 years higher than that of rural women. As age increases, the urban–rural gap in life expectancy decreases. The absolute changes in DFLE for those aged 60 is 1.637 years for men and 1.454 years for women. At the age of 85, these changes decrease to 0.239 years for men and 0.682 years for women.

**Figure 4:**
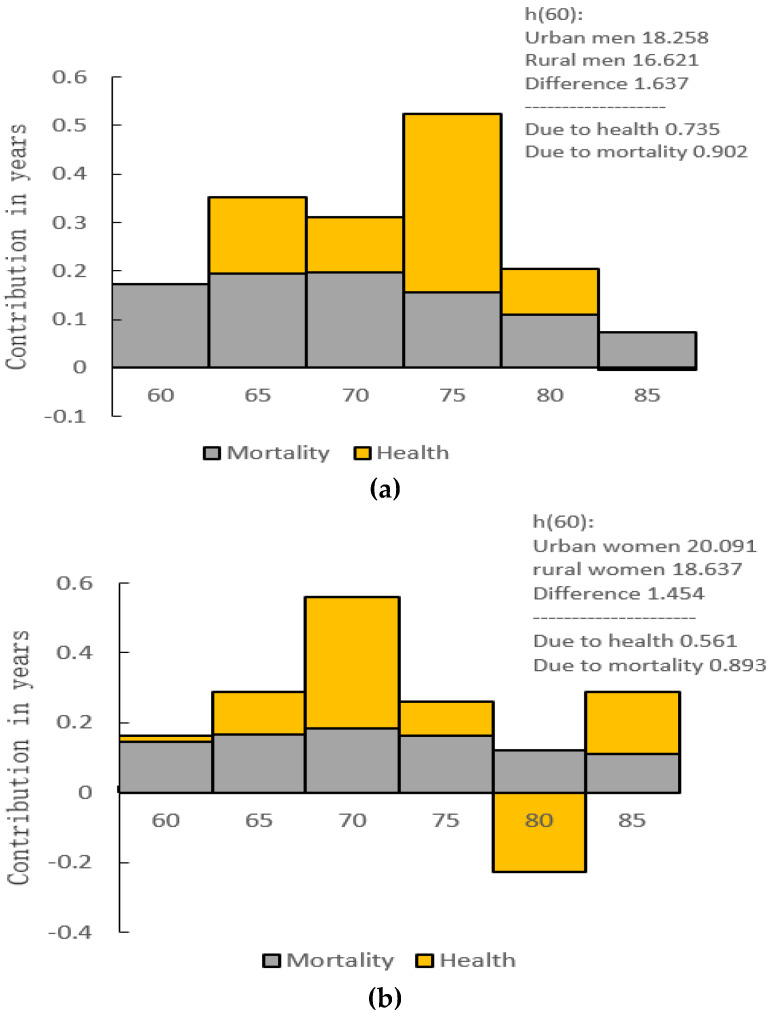
Decomposition of the difference between the DFLE of elderly in Jiangxi Province at age 60 between urban and rural areas. (**a**) men (**b**) women.

Figure 4 shows that the difference in DFLE between urban and rural men over 60 years is 1.637 years, of which 0.902 years are attributable to the mortality rate, and 0.735 years are due to the disability-free prevalence (the 75-year-old group has the largest contribution at 0.369 years). In addition, the difference in DFLE between urban and rural women over 60 years old is 1.454 years; of which 0.893 years are attributable to the mortality rate, and 0.561 years are due to the disability-free prevalence (the 70-year-old group has the largest contribution of 0.374 years).

## 4. Discussion

As in previous studies [17,18,35,36], we analyzed the DFLE and its proportion of the elderly over 60 in Jiangxi Province by sex differences. From the results, we found some similarities and differences compared with the previous research. Similarly, women’s DFLE is higher than that of men in all age groups (except at 85 years old), and the DFLE/LE is lower than that of men. Through the decomposition method, we found that the gender difference in DFLE of the elderly over 60 is mainly due to mortality, and the disability-free prevalence reduced the gap, which also suggested that reducing the mortality difference can narrow most of the DFLE gaps between genders. The values of LE, DFLE, and DLE are slightly different from these studies in other countries or in different provinces in China, which may be affected by sampling errors, different sampling methods, sample sizes, different evaluation standards, culture, and economics [37]. As the age increases, DLE increases as well, while DFLE and DFLE/LE gradually decrease, and the decreasing trend of the DFLE is parallel to that of LE. It is worth noting that in the gender comparison of DFLE, only the 60-, 65-, and 70-year-old groups have statistically significant differences. There is no statistically significant gender difference in the age group after the age of 75, which may be due to the limited sample size. Previous studies [26,38,39] have shown that compared with men, women have an advantage in survival, but at the same time, they spend more years in worse health. Specifically, older women in Jiangxi Province have obvious advantages in LE and DFLE. However, the DFLE/LE of women is lower than that of men, which means the time of being unable to take care of themselves in their later lives is longer than that of men. Therefore, compared with the quality of life between the genders, women’s health status is at a disadvantage.

Taking the regional differences into account, we conducted a discussion based on urban and rural areas. For DFLE, the gender difference between urban and rural areas is roughly the same as the whole population. Both urban and rural women’s DFLE are higher than for men. It is worth noting that the DFLE of urban or rural women in the 85-year-old group is lower than that of men, but the difference has no statistical significance. For DFLE/LE, the gender differences in different areas are similar to the whole population; urban and rural men’s figures are higher than for women. Comparing the DFLE/LE of urban and rural men and women together, the ratio of urban men is the highest in each age group, and the ratio of rural women is the lowest in the 60-year-old age group (85.3%). However, the ratio of rural women over 80 years old is abnormal, and it is better than that of urban women aged 80. It can be seen that with the Jiangxi Province divided into urban and rural dimensions, there are severe problems in the life quality of elderly rural women in their later years. Their life quality is the poorest among the four groups. More than half of them cannot take care of themselves after age 85. Therefore, we argue that rural older women’s terrible life quality is more likely to be attributed to their lower socioeconomic status and educational level. Relevant research [14,40] showed that people with different education and economic levels had differences in chronic diseases, health behaviors, and preventive healthcare use. These were considered as risk factors for functional disability, and people with higher education levels performed better in these aspects.

With the increase of age, the absolute change in LE and DFLE of urban and rural men is increasing while the magnitude is gradually shrinking. The increase in LE is smaller than the DFLE gain (except at age 85) which indicates that rural men have more time with disability than urban men do, and the situation is more serious at a young age. Using the decomposition method to calculate the contributed years caused by the mortality and disability-free prevalence, we found that the difference was due more to the mortality rate, but the impact of the disability-free prevalence could not be ignored. The 75-year-old group’s disability-free prevalence contributed even more than the mortality, which suggested that when narrowing the urban–rural mortality gap, attention should also be paid to the disability-free prevalence of the elderly. The two aspects should be combined to reduce the DFLE difference. Our results are similar to Islam’s research [7]. Another researcher showed that rural men in China have more accesses to engage in physical labor, and regular exercise, which can help reduce the risk of disability [41], but this conclusion is not reflected in our results.

The absolute change in the life expectancy of urban–rural women is similar to that of men in general, but it is greater for women than men in terms of all age groups. For DFLE, the absolute difference is higher than the difference in life expectancy (except at ages 75 and 80) which means that rural women spend more time with disability than urban men do. The results from the decomposition method show a similarity between the urban–rural men and women. The only slight difference is that the contribution of women’s disability-free prevalence in the 70-year-old group is greater than that of the mortality. With more job opportunities, social resources, medical resources, etc., in cities, young people tend to be attracted to these areas easily [37]. In China, many of the elderly may continue to live alone in the countryside as their children move into the city, resulting in a large number of empty-nest elderly adults; rural women in particular [42]. It is worth noting that the performance of rural women in the 80-year-old group is more prominent with the DFLE/LE being even higher than that of urban women, and at the same time, the disability-free rate of rural women in this age group is also higher than that of urban women. In Figure 4, due to this special situation, the DFLE difference of DFLE between urban–rural women over 60 years old was reduced.

The prevalence of disability has increased in most administrative divisions and the country as a whole [1]. In less developed regions, there is still considerable social and health inequality and poor access to health care. Describing and addressing the health inequality of the elderly are key focuses of China’s public health agenda [6]. In Jiangxi Province, more attention should be paid to the health of elderly women, their health needs should be reasonably evaluated, and sufficient resources such as medical resources and elderly care resources should be allocated. Considering the urban–rural differences, rural elderly; especially rural women, have become vulnerable groups in our study. The government should take actions to reduce transport inequality between urban and rural areas, provide them with necessary health services, and ensure that they can acquire medical services and enjoy health resources fairly. Furthermore, the whole province should pay attention to the education of rural people, improve their education level, create income opportunities to eliminate regional inequality, and reduce mortality and disability.

Finally, some limitations should be mentioned in this study. First, the ADL scale (8 items) used in this study is different from the ADL scale used in most studies, and there may be certain deviations when making comparisons with similar studies. Compared with the ADL scale of 6 items, the disability-free rate may be lower, while compared with the scale combined with IADL, the disability-free rate may be higher. Second, the difference for the elderly at an advanced age is relatively poor in regularity. Z test had been done for the DFLE comparisons of the two pairs. Most of the differences in the comparison of the elderly at an advanced age were not considered statistically significant. It can be seen that the sample had an important effect on the research. Last, the results of this study are influenced by economic and demographic factors, and as Jiangxi Province is at the middle level in the whole country, our results can be used as a reference for the national average and provinces with similar economic and demographic situations, but they cannot be generalized to all provinces in China. Nevertheless, these data from the Sixth Health Service Survey and used in this study, clearly described the gender differences and urban–rural differences in DFLE in Jiangxi Province in 2018. The influencing factors of DFLE in Jiangxi Province also need to be further studied.

## 5. Conclusions

Although the increase in DFLE is accompanied by the increase in LE, the life quality of the elderly population has not increased, especially for women. When considering regional differences, rural elderly, especially rural women, experience more years with disability and a lower life quality in their remaining life. They need to receive more attention and be provided more care from the government sector and the department of health.

## Figures and Tables

**Table 1:** LE, DFLE, and the proportion of DFLE in Jiangxi Province, China, by sex and selected ages for the year 2018.

Age	Men				Women			
	LE	DFLE	95%CI	DFLE/LE	LE	DFLE	95%CI	DFLE/LE
60	19.127	17.157	(16.837, 17.476)	0.897	22.027	19.055	(18.624, 19.487)	0.865
65	15.369	13.578	(13.259, 13.898)	0.883	17.893	15.014	(14.572, 15.456)	0.839
70	12.019	10.297	(9.961, 10.633)	0.857	14.103	11.222	(10.757, 11.686)	0.796
75	9.209	7.492	(7.122, 7.862)	0.814	10.803	7.934	(7.437, 8.431)	0.734
80	6.864	5.240	(4.826, 5.654)	0.763	8.006	5.299	(4.747, 5.850)	0.662
85	5.134	3.496	(2.943, 4.049)	0.681	5.851	3.060	(2.350, 3.770)	0.523

LE, Life expectancy; DFLE, Disability-free life expectancy; 95% CI, 95% confidence intervals.

**Table 2:** LE, DFLE, and the proportion of DFLE for selected ages in urban and rural areas.

Age	Men					Women			
	LE	DFLE	95%CI	DFLE/LE		LE	DFLE	95%CI	DFLE/LE
					Urban				
60	20.068	18.258	(17.837, 18.680)	0.910		23.102	20.091	(19.487, 20.694)	0.870
65	16.200	14.599	(14.187, 15.011)	0.901		18.859	15.952	(15.341, 16.562)	0.846
70	12.725	11.151	(10.719, 11.584)	0.876		14.947	12.011	(11.371, 12.651)	0.804
75	9.776	8.206	(7.740, 8.672)	0.839		11.502	8.418	(7.722, 9.113)	0.732
80	7.305	5.629	(5.085, 6.172)	0.771		8.569	5.617	(4.851, 6.384)	0.656
85	5.448	3.705	(3.000, 4.409)	0.680		6.284	3.456	(2.487, 4.425)	0.550
					Rural				
60	18.957	16.621	(16.091, 17.151)	0.877		21.837	18.637	(17.944, 19.331)	0.853
65	15.221	13.032	(12.490, 13.574)	0.856		17.724	14.607	(13.897, 15.317)	0.824
70	11.895	9.818	(9.245, 10.392)	0.825		13.957	10.890	(10.144, 11.636)	0.780
75	9.111	7.036	(6.390, 7.683)	0.772		10.684	7.853	(7.071, 8.636)	0.735
80	6.788	5.076	(4.363, 5.789)	0.748		7.912	5.328	(4.458, 6.197)	0.673
85	5.081	3.465	(2.476, 4.454)	0.682		5.780	2.774	(1.642, 3.906)	0.480

LE, Life expectancy; DFLE, Disability-free life expectancy; 95% CI, 95% confidence intervals.

## Data Availability

Not applicable.
